# Replicating the EnhanceFitness Physical Activity Program in Hawai`i’s Multicultural Population, 2007-2010

**DOI:** 10.5888/pcd9.110155

**Published:** 2012-03-22

**Authors:** Michiyo Tomioka, Naomi Sugihara, Kathryn Braun

**Affiliations:** University of Hawai`i at Mānoa, Office of Public Health Studies, John A. Burns School of Medicine; County of Kaua`i Agency on Elderly Affairs, Honolulu, Hawai`i; Office of Public Health Studies, John A. Burns School of Medicine, and Myron B. Thompson School of Social Work, University of Hawai`i at Mānoa, Honolulu, Hawai`i

## Abstract

**Background:**

Despite evidence of the benefits of regular physical activity, many older adults are not physically active. Health professionals are challenged to replicate evidence-based programs to address low levels of physical activity among members of their communities.

**Community Context:**

EnhanceFitness is an evidence-based group exercise program developed in Seattle to increase the strength, flexibility, and balance of older adults. Hawai`i's Healthy Aging Partnership supported the rural island of Kaua`i to select, adapt, implement, and evaluate EnhanceFitness to increase physical activity among older adult residents (75% Asian/Pacific Islander [API]).

**Methods:**

Evaluation measures of the replication of EnhanceFitness included fidelity of EnhanceFitness delivery and participants' attendance, satisfaction with the program, confidence to exercise regularly, and pre-post fitness check measures of physical performance (chair stands, arm curls, and the up-and-go test).

**Outcomes:**

Between July 2007 and December 2010, 223 Kaua`i residents enrolled in EnhanceFitness; 178 (80%) participated at least 4 months and completed the 4-month fitness checks. EnhanceFitness classes were offered with a high degree of fidelity, and both API and white participants significantly improved their physical performance (chair stands, *t* = −11.06, *P* < .001; arm curls, *t* = −6.66, *P* < .001; and up-and-go test, *t* = 6.56, *P* < .001). Participants reported high satisfaction with the program and instructors and high confidence to continue to exercise regularly.

**Interpretation:**

EnhanceFitness is replicable in Hawai`i and increased physical performance among API and white older adults. This case study outlines a replication process that other communities can follow.

## Background

Regular physical activity can promote physical and psychological well-being and reduce risk of disability and vulnerability to chronic diseases among older adults (1). However, despite evidence supporting benefits of regular physical activity, many older adults are not physically active (2). Health problems, fear of falling, and inconvenience are common reasons cited for being physically inactive. Designing physical activity programs for older adults and improving access to these programs are key strategies to encourage older adults to become more physically active (3).

To support well-being among older adults, attention has been given to implementing evidence-based health promotion interventions that have proved to be effective in reducing risk of disability and chronic diseases (4). Such evidence-based programs, now being widely disseminated with support from federal agencies, focus on physical activity, chronic disease self-management, mental wellness, and healthy eating (5).

Although there is push for broad replication of evidence-based programs, translating evidence-based interventions from the study site to other communities can be challenging. Barriers include insufficient training for providers in these programs, limited time and resources of providers to offer new programs, and inadequate organizational infrastructure and systems to support translation (6).

## Community Context

The state of Hawai`i has 4 counties (Kaua`i, Honolulu, Maui, Hawai`i). More than 20% (approximately 277,000) of Hawai`i's residents are older adults (aged ≥60 years), and this percentage is expected to increase to 25% by 2030. More than 75% of the aging population in Hawai`i is Asian, Native Hawaiian, and Pacific Islander (7). Approximately 22% of the state's older adults report difficulty walking, climbing stairs, reaching, lifting, or carrying, and more than 55% do not meet recommended physical activity levels (8). These statistics are similar across counties.

Kaua`i County has approximately 13,800 (21%) older adults. Of these older adults, 53% are Asian/Pacific Islander (API), 34% report at least 1 disability, and 60% experience loss of physical performance (9). Kaua`i County is rural and is challenged by limited health promotion opportunities for older adults (7). In 2003, Kaua`i County began searching for programs to improve physical activity among its older residents.

Hawai`i's Healthy Aging Partnership is a statewide coalition that was formed in 2003 to improve the well-being of older adults by replicating evidence-based health promotion programs. Coalition members included professionals from government offices for aging and public health, eldercare agencies, and the University of Hawai`i at Mānoa. Representatives from the County of Kaua`i Agency on Elderly Affairs were founding members of the partnership. Early in the partnership, all 4 counties were supported to assess health promotion preferences of their older adults, and all counties piloted small exercise programs. Successful completion of these activities helped Hawai`i secure federal grants in 2006 to replicate 2 evidence-based programs in the state, and Kaua`i County chose to replicate EnhanceFitness (10).

EnhanceFitness is a group exercise program developed by Senior Services in Seattle to increase the strength, flexibility, and balance of older adults through structured group exercise sessions of stretching, low-impact aerobics, balance, and strength training (11). The program, lead by certified fitness instructors trained in EnhanceFitness, is a 1-hour class held 3 times per week. EnhanceFitness is effective in improving physical performance of older adults in different communities and among various ethnic groups in the United States (11-13).

Research that documents cross-community translation of evidence-based programs and explores their effectiveness in new populations and locations can help in the dissemination and use of evidence-based programs. The purpose of this article is to describe how Kaua`i County selected, adapted, implemented, and evaluated EnhanceFitness to increase availability of physical activity programs and physical performance among older adults. Evaluation measures included fidelity of EnhanceFitness delivery and participant attendance, satisfaction, confidence to exercise regularly, and pre-post fitness check measures of physical performance (chair stands, arm curls, and the up-and-go test). We assessed the effectiveness of EnhanceFitness among API and white participants.

## Methods

### Program adaptation tools

To adapt EnhanceFitness to Kaua`i County, the Hawai`i Healthy Aging Partnership used the Track Change Tool adapted by the National Council on Aging from Peterson (14) to deconstruct the program into aspects or phases (ie, marketing, recruitment, staffing, training, scheduling, and evaluation). We noted how each aspect of the program was carried out in its original offering in Seattle and then provided a detailed description about how each aspect would be implemented in Kaua`i County. This tool ensured that adapters thoroughly planned the replication and identified possible discrepancies in implementation compared with the original program.

We then used the "adaptation traffic light" to judge the discrepancies because some elements of an evidence-based program should not be modified (15). Red-light changes are those that cannot be made, such as substantially shortening the program or deleting activities. Green-light changes, such as creating a local name for the program, can be freely made. Yellow-light changes are those that can be made with caution because there is a chance that they could decrease program effectiveness; yellow-light changes are recommended to be reviewed with the original developers of the evidence-based program (15).

### Fidelity monitoring measures

To ensure that key components of EnhanceFitness were delivered exactly as prescribed by the program developers, Kaua`i County recruited qualified trainers and instructors, arranged training in EnhanceFitness, and monitored fidelity. An EnhanceFitness master trainer, who is certified to provide instructor training, monitor fidelity of program delivery, and offer Enhance Fitness classes, regularly monitored EnhanceFitness instructors by using a fidelity monitoring tool developed and validated by Seattle's Senior Services (16). The master trainer rated instructors on 8 fidelity items using 3 categories: above standard, meets standards, and needs improvement. Monitoring occurred in the first week of the class and then every 4 months thereafter. After every evaluation, recommendations for instructor improvement were provided.

### Evaluation measures and analysis

**Demographics**

At enrollment, participants completed consent, registration, and health forms and provided physician clearance for participation; Senior Services (16) provided these forms. The registration form solicited demographic characteristics (age, sex, and ethnicity), English-language proficiency, and self-reported chronic conditions. Because we intended to test the effectiveness of EnhanceFitness with rural-dwelling API residents of Kaua`i, we compared outcomes of API participants with those of white participants. Therefore, we excluded the outcomes of 3 participants who were not either API or white. The final sample for analysis was 223 participants who enrolled between 2007 and 2010, including 177 API and 46 white participants. Attendance was collected using a form from Senior Services (16).

**Fitness checks**

Physical performance refers to a person’s lower- and upper-body strength, agility, and balance. Fitness checks, tests developed by Senior Services (16) to assess physical performance, were 1) chair stands, in which the number of times the participant can move from sitting to standing in 30 seconds was recorded; 2) arm curls, in which the number of times the participant can lift a weight (5-pound weight for women, 8-pound weight for men) in 30 seconds was recorded; and 3) the up-and-go test, in which the number of seconds to stand, travel 8 feet, round a cone, return to the chair, and be reseated was recorded. During the fitness checks, the older adult was asked how many times he or she had fallen in the past 4 months. Participants completed fitness checks every 4 months. We used paired t tests to analyze within-group improvement by comparing fitness check data at baseline and at 4 months.

**Satisfaction**

We gathered data on participant satisfaction by using a tool adapted from Senior Services (16), which was administered at the 4-month fitness checks and annually thereafter. The survey asked how program participants heard about EnhanceFitness (county agency, service providers, doctors/health care professionals, mass media [newspaper, brochure, television, or radio], or other), their reasons for enrollment (wanted to improve health, wanted to meet with friends, wanted to learn about exercise, or other), and their satisfaction and confidence to exercise regularly. We assessed the satisfaction and confidence items using a scale from 1 to 10 (1 being "not at all" and 10 being "totally"). The satisfaction survey was anonymous, and 119 participants attending for more than a year completed more than 1 survey. Therefore, 374 satisfaction forms were available for analysis.

## Outcomes

### Program adaptation

From the EnhanceFitness training and manual, we identified several red-light changes, such as shortening the duration of the exercise program, eliminating phases of exercise (eg, the warm-up), and adjusting the tempo of the music for each phase of exercise. These types of changes were not attempted (Table 1). Green-light changes included using familiar music that met the tempo requirements (for example, Hawaiian music for the warm up) and renaming certain exercises to relate them to daily activity (for example, the exercise called "stepping over an object" was renamed "getting in and out of the bathtub").

In trying to replicate the program, we identified 2 potential yellow-light changes that were discussed with Senior Services. In Seattle, EnhanceFitness was offered in indoor, temperature-controlled, exercise rooms with a carpet-free wooden floor and straight-back chairs. However, many of the community-based facilities on Kaua`i were built with concrete floors and trade-wind ventilation and offered only folding chairs. Senior Services agreed that our classes could be held in these facilities but recommended purchasing straight-back chairs when funds allowed. We also asked if our program participants could wear *zori* (ie, flip-flops) or sandals during the program, as some did not own closed-toe athletic shoes. However, Senior Services strongly recommended against this change, and the Hawai`i Healthy Aging Partnership agreed to find resources for older adults in need of financial assistance with purchasing appropriate shoes.

Once the adaptations were set and implementation planned, representatives from Kaua`i County organized and coordinated the classes, finding certified fitness instructors willing to be trained as EnhanceFitness master trainers and instructors, scheduling training, securing sites, and recruiting participants. Hawai`i's Healthy Aging Partnership helped to bring EnhanceFitness trainers from Senior Services, adapt marketing tools, develop consent forms, obtain institutional review board approval, monitor fidelity of delivery, and evaluate participant outcomes.

### Fidelity of EnhanceFitness delivery

EnhanceFitness instructors must have nationally recognized fitness instructor certification, and our initial group of certified fitness instructors was trained by Senior Services in June 2007. By January 2011, 5 trainings had been provided, yielding 1 master trainer and 21 instructors on Kaua`i. However, by 2011, only 8 trained instructors were offering classes regularly (7 were not interested in offering classes regularly, 4 had left Kaua`i, and 3 had found full-time jobs). Our results showed that all instructors had areas in need of improvement during their first week of offering EnhanceFitness. At month 4, however, instructors met or exceeded standards in all 8 areas, suggesting that EnhanceFitness was being offered with a high degree of fidelity.

**Participants**

Initially, Kaua`i County implemented EnhanceFitness classes 3 times per week every week of the year at 2 sites. The average class size was 19.3 (range, 15-21). EnhanceFitness classes were popular, and new sites were opened in 2008, 2009, and 2010. By 2011, 8 different classes were offered at 7 sites in Kaua`i County.

Compared with white participants, API participants were older, more likely to live in larger households and to have lower incomes, and more likely to have hypertension (Table 2). There were more men among the API participants. More than half (60%) of the EnhanceFitness participants attended classes at least twice per week.

Of the 223 initial enrollees, 178 (80%) completed the 4-month fitness check, and 119 (53%) attended longer than 1 year (Figure). Participants dropped out because they were sick, moved, or found that EnhanceFitness classes conflicted with other activities.

**Figure.**
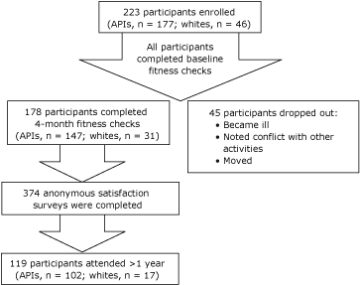
Number of program participants at each phase, EnhanceFitness, Kaua`i County, Hawai`i, 2007-2010. The anonymous satisfaction survey was administered at the 4-month fitness checks, with the assessment of physical performance, and annually thereafter (119 participants who attended the program for more than 1 year completed more than 1 satisfaction survey). Abbreviation: APIs, Asians/Pacific Islanders.

**Fitness checks**

All program participants (N = 223) successfully completed baseline fitness checks during the first week of class, and 178 (80%) participants completed the 4-month fitness checks (Table 3). Both API and white participants realized significant improvement in the 3 performance measures and a nonsignificant reduction in self-reported falls in the previous 4 months.

**Program satisfaction**

Because the anonymous satisfaction survey was administered at the 4-month fitness checks and annually thereafter, 374 forms were completed. More than one-third of EnhanceFitness participants heard about the program through county agency on aging (33%, n = 124) and partner agencies (21%, n = 78). Almost all participants (97%) attended EnhanceFitness because they sought to improve their health. Other reasons included desire to learn about exercise (44%, n = 164) and meet with friends (35%, n = 130). Program participants were very satisfied with EnhanceFitness and the program's instructors (mean, 9.51, standard deviation [SD] = 0.93), willing to continue the exercise learned from EnhanceFitness (mean, 9.22, SD = 1.43), and confident that they could continue to exercise regularly (mean, 9.18, SD = 1.40). We were not able to assess differences between API and white participants because of the anonymity of the survey.

## Interpretation

Evidence-based physical activity programs are available, but the challenges in translating them to community practice are documented (6,17). EnhanceFitness has expanded in various places with diverse groups, yet few studies have been published about its effectiveness (11-13). Hawai`i offers a unique environment to study replication and effectiveness of evidence-based programs because of its culturally diverse population.

In replicating EnhanceFitness, we found that few adaptations were needed. As others have found, the use of familiar music helps "localize" EnhanceFitness (12), and the names of exercises can be changed. We requested and were granted allowances on the types of facilities where EnhanceFitness could be offered. Apart from this, no adaptations were needed to increase the community appropriateness of EnhanceFitness. Older adults who attended regularly embraced the exercise program.

Although EnhanceFitness did not require major adaptations to fit our older adults, the replication process was assisted by the Track Changes Tool and the "adaptation traffic light." Using these tools forced us to carefully consider how EnhanceFitness had been implemented by its designers and how closely we could replicate it in Kaua`i County. Other communities would benefit from guidelines on using these tools and from a list of allowable and unallowable modifications to EnhanceFitness.

Hawai`i is among many US states that are participating in a nationwide effort to replicate evidenced-based health promotion programs for older adults. Therefore, we were able to compare our data with those of other states. We initially were disappointed by our 4-month fitness check completion rate (80%) and the proportion of people attending the class more than twice each week (60%). However, these figures compared favorably to the national average of 60% completing the 4-month fitness check and 39% attending the class more than twice each week, based on 3,803 EnhanceFitness enrollees across 7 states in 2009 (18). Our program participants were older (mean age, 78 y) than participants from other studies that assessed the effectiveness of EnhanceFitness (mean age, 72 y); however, our pre-post findings were similar to those from Senior Services (12), which showed increases in chair stands (from 12.7 to 14.3, *P* < .001), and arm curls (from 16.9 to 19.4, *P* < .001), and reduced time for the up-and-go test (from 7.5 to 7.1 seconds, *P* < .001) (11,13). Similarly positive outcomes also reflect that EnhanceFitness was offered in Hawai`i with good fidelity, confirming the value of ongoing fidelity monitoring by the master trainer.

States planning to implement EnhanceFitness should expect that some certified fitness instructors trained in EnhanceFitness may not be able to follow through with leading classes. We lost 13 instructors from our pool because they lost interest, changed jobs, or moved away from the county. An issue for the Hawai`i EnhanceFitness is that we have only 1 recognized master trainer who believes in and is committed to the program. Because providing transportation for this master trainer to other counties to train instructors and monitor program fidelity, expansion of EnhanceFitness to other communities would require us to develop more master trainers and committed instructors.

Efforts to replicate evidence-based programs to serve a state's aging population can be challenging (10). There is a need for the Hawai`i Healthy Aging Partnership to help increase the capacity of Hawai`i's service providers to find and replicate evidence-based programs. Attention to capacity building at the agency level can facilitate program implementation and sustainability (19).

Another challenge to replicating evidence-based programs is sustaining them. In the case of Hawai`i, funds to support the replication of EnhanceFitness were provided by the US Administration on Aging, National Council on Aging, county government, and US Department of Health and Human Services.

Hawai`i's Healthy Aging Partnership analyzed costs associated with starting EnhanceFitness and estimated potential cost savings to Hawai`i that may have resulted from the implementation of EnhanceFitness. Our analysis was based on a comprehensive controlled study of EnhanceFitness conducted in Seattle that showed that participants who attended at least 1 EnhanceFitness class per week cost the health care system 20% less per year than controls (20). From this information, we estimated that the expected investment-to-return ratio for EnhanceFitness on Kaua`i was 1:1.8 (21). However, we have not been successful in persuading insurers to reimburse EnhanceFitness.

Our research had limitations. First, participant data were collected by instructors and program staff; although they were trained to conduct each physical performance test, individual deviations may have occurred. Second, falls were self-reported and subject to recall bias. Third, we did not have a control group for this study.

Replicating an evidence-based program requires effort; programs must be identified to fit to new communities, provider capacity must be built, and the programs must be implemented and sustained. However, our study findings indicate that EnhanceFitness is capable of being modified for and is effective in an API community. This case study outlines a replication process that other communities can follow.

## Figures and Tables

**Table 1.** Hawai`i Modifications to the EnhanceFitness, Using Light Grading System^a^

Modification	Light Grading	Description
Using local ethnic music	Green	Using program participants' familiar music increased motivation for program participation.
Using different names for certain exercises	Green	We renamed certain exercises to relate them to daily activity (eg, the exercise called "stepping over an object" was renamed "getting in and out of the bath tub").
Holding classes on concrete floors	Yellow	Facilities in Hawai`i are built with concrete floors with trade-wind ventilation. Senior Services "approved" our use of these facilities.
Using folding chairs	Yellow	Facilities had only folding chairs. Senior Services allowed us to use these chairs but recommended purchasing straight-back chairs when funds allowed.
Using different shoes	Yellow→ Red	Older adults in Hawai`i usually wear *zori* (ie, flip-flops) or sandals, and some do not own closed-toe athletic shoes. Senior Services felt strongly that zori and sandals were unsafe, and the Hawai`i Healthy Aging Partnership established a fund to purchase proper shoes for participants who could not afford them.

a A traffic light system was used to judge the discrepancies. Red-light changes are those that cannot be made. Green-light changes can be freely made. Yellow-light changes are those that can be made with caution, and it is recommended that yellow-light changes be reviewed with the original developers of the evidence-based program.

**Table 2.** Participants' Demographic Characteristics, Kaua`i County, 2007-2010 a

Characteristics	Total (n = 223)	API (n = 177)	White (n = 46)
**Age, mean (range), y**	78.2 (61-95)	78.8 (61-94)	76.0 (62-95)
60-74	76 (34.1)	54 (30.5)	22 (47.8)
75-84	93 (41.7)	79 (44.6)	14 (30.4)
≥85	54 (24.2)	44 (24.9)	10 (21.7)
**Sex**			
Male	14 (6.3)	8 (4.5)	6 (13.0)
Female	209 (93.7)	169 (95.5)	40 (87.0)
**Ethnicity**			
White	46 (20.6)	NA	46 (100)
Japanese	115 (51.6)	115 (65.0)	NA
Filipino	45 (20.2)	45 (25.4)	NA
Native Hawaiian/Pacific Islander	17 (7.6)	17 (9.6)	NA
**Marital status**			
Married	94(42.2)	73 (41.2)	21 (45.7)
Divorced	20 (9.0)	12 (6.8)	8 (17.4)
Widowed	103 (46.2)	87 (49.2)	16 (34.8)
Other (single, partnered)	6 (2.6)	5 (2.8)	1 (2.2)
**Household size, mean no. (range)^b^**	1.97 (1-7)	1.99 (1-7)	1.89 (1-5)
1	84 (37.8)	69 (39.2)	15 (32.6)
2	96 (43.2)	73 (41.5)	23 (50.0)
>2	42 (18.9)	34 (19.3)	8 (17.4)
**Income, $^c^**			
<15,000	70 (32.1)	62 (35.8)	8 (17.8)
15,000-24,999	68 (31.2)	53 (30.6)	15 (33.3)
25,000-49,999	49 (22.5)	38 (22.0)	11 (24.4)
50,000-75,000	22 (10.1)	17 (9.8)	5 (11.1)
>75,000	9 (4.1)	3 (1.7)	6 (13.3)
**Have English limitation^d^**	14 (6.3)	10 (5.6)	4 (8.7)
**Disabled^b^**	29 (13.0)	23 (13.0)	6 (13.0)
**Chronic condition**			
Diabetes	51 (22.9)	40 (22.6)	11 (23.9)
Heart Diseases	31 (13.9)	23 (13.0)	8 (17.4)
Hypertension	107 (48.0)	90 (50.8)	17 (37.0)
Lung Disease	12 (5.4)	10 (5.6)	2 (4.3)
Cancer	21 (9.4)	15 (8.5)	6 (13.0)
Depression	16 (7.2)	8 (4.5)	8 (17.4)
Arthritis	89 (39.9)	72 (40.7)	17 (37.0)

Abbreviation: API, Asian/Pacific Islander; NA, not applicable.

a All values are given as n (%), unless otherwise indicated.

b One API respondent had missing data for this category.

c Four API respondents and 1 white respondent had missing data for this category.

d Two API respondents had missing data for this category.

**Table 3:** Participants' (n = 178)^a^ Baseline and 4-Month Fitness Checks Results, Kaua`i County, 2007-2010

Item	Mean (Standard Deviation)			*t*	*df*	*P* Value

	Baseline	4 Months	Change			
**Chair stand^b^ repetitions**	11.52 (3.90)	11.45 (4.01)	−2.93 (3.53)	−11.06	176	.001
APIs	11.67 (4.14)	14.51 (4.20)	2.84 (3.76)	9.11	145	.001
Whites	10.81 (2.34)	14.19 (3.03)	3.39 (2.11)	8.95	30	.001
**Arm curl repetitions**	10.66 (3.55)	12.63 (3.78)	1.97 (3.94)	6.66	176	.001
APIs	10.27 (3.46)	12.22 (3.51)	1.95 (3.91)	6.03	145	.001
Whites	12.52 (3.41)	14.58 (4.42)	2.07 (4.13)	2.78	30	.009
**Up-and-go,^c^ seconds**	8.54 (3.56)	7.54 (3.11)	−0.99 (2.02)	−6.56	177	.001
APIs	8.82 (3.75)	7.80 (3.30)	−1.02 (2.14)	−5.79	146	.001
Whites	7.23 (2.06)	6.35 (1.54)	−0.87 (1.36)	−3.57	30	.001
**Number of falls**	0.20 (0.55)	0.12 (0.38)	−0.08 (0.64)	−1.24	101	.22
APIs	0.19 (0.56)	0.12 (0.39)	−0.07 (0.68)	−0.95	85	.35
Whites	0.25 (0.45)	0.13 (0.34)	−0.13 (0.34)	−1.46	15	.16

Abbreviation: df, degrees of freedom; APIs, Asians/Pacific Islanders.

a Of total respondents, 147 were API and 31 were white.

b Exercise in which the number of times the participant can move from sitting to standing in 30 seconds was recorded.

c Exercise in which the number of seconds it takes the participant to stand, travel 8 feet, round a cone, and return to the chair was recorded.
